# Sandifer Syndrome Case Report: An Unusual Presentation with Paroxysmal Torticollis

**DOI:** 10.3390/reports8010029

**Published:** 2025-03-10

**Authors:** Giorgia Segre, Martina Penzo, Giovanna Zorzi, Federica Graziola

**Affiliations:** 1 Department of Pediatric Neurosciences, Fondazione IRCCS Istituto Neurologico Carlo Besta, 20133 Milan, Italy; 2 Department of Health Sciences, University of Milan, 20122 Milan, Italy

**Keywords:** Sandifer syndrome, paroxysmal torticollis, abnormal head posture, gastroesophageal reflux disease, paroxysmal nonepileptic events

## Abstract

**Background and Clinical Significance**: Sandifer syndrome is an uncommon manifestation of gastroesophageal reflux disease, characterized by paroxysmal episodes of abnormal posturing, particularly involving the neck and upper body, often associated with underlying esophageal discomfort. **Case Presentation**: In this report, we present a 16-month-old infant who exhibited multiple daily paroxysmal episodes of atypical head posturing, primarily tilted to the right, each lasting less than 10 s, with spontaneous resolution. Notably, these episodes lacked other neurological or systemic symptoms, and the clinical presentation differed from classical descriptions of Sandifer syndrome, which often include more prolonged dystonic posturing or correlation with feeding. The diagnosis was supported by the resolution of symptoms following the administration of a proton pump inhibitor, highlighting the importance of recognizing this condition in infants with unexplained posturing behaviors. **Conclusions**: This case emphasizes the variability in clinical manifestations of Sandifer syndrome and underscores the need for a high index of suspicion, as timely management of gastroesophageal reflux disease can lead to complete symptom resolution and prevent unnecessary neurological investigations.

## 1. Introduction and Clinical Significance

Gastroesophageal reflux in the pediatric population presents with a wide range of symptoms, often mimicking neurological or psychiatric conditions [1]. Sandifer syndrome (SS) is a rare manifestation of gastroesophageal reflux disease (GERD) and hiatal hernia, characterized by abnormal spastic torticollis, dystonic movements, and severe hypotonia [2]. First described by Kinsbourne in 1964, based on Sandifer’s observations [1,3], SS predominantly affects children aged 18–36 months. Although its exact prevalence is unknown, SS is estimated to affect less than 1% of children with GERD [4,5].

The pathophysiology remains incompletely understood, though dysfunction of the lower esophagus is thought to play a key role [6]. Neck posturing may help clear acid from the esophagus, providing symptom relief as a result from vagal reflexes involving the nucleus tractus solitarii (NTS) and accessory nerve pathways [7].

Diagnosing SS is challenging due to its atypical presentation [8], often leading to misdiagnosis as epilepsy or other neurobehavioral disorders and unnecessary treatments. Early recognition is crucial to prevent such interventions [9]. Lifestyle modifications are the first-line treatment for GERD and SS, including upright positioning post-feeding, prone positioning, and hypoallergenic formulas for suspected cow’s milk protein allergy. If symptoms persist, pharmacological therapy with acid suppressants (histamine—H2 receptor antagonists, proton pump inhibitors—PPIs), buffers (antiacids), or prokinetics is considered [10]. Surgical intervention, such as fundoplication, may be required in refractory cases [2].

Despite the availability of treatment options, reports on SS have been infrequent: only a few systematic reviews have recently been published on this subject [11,12]. Thus, the present report aims to improve the understanding and management of this rare disorder by sharing our clinical experience.

## 2. Case Presentation

### 2.1. Clinical Presentation and History

**Figure 1:**
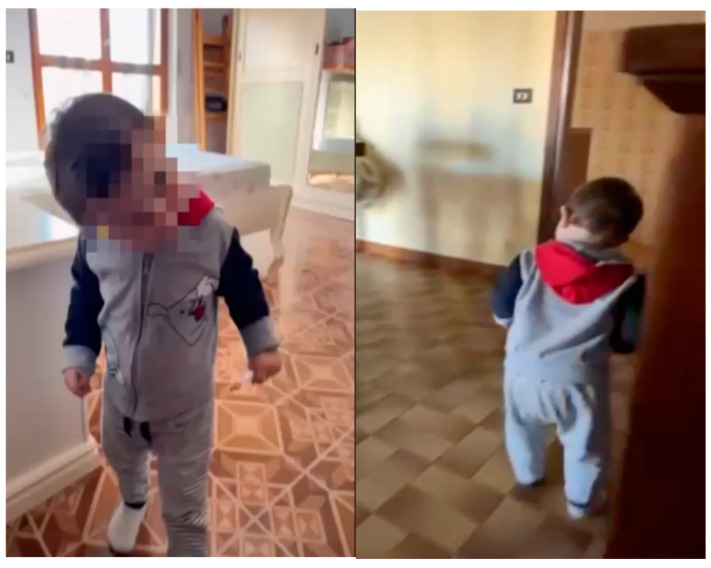
Presentation of paroxysmal torticollis prior to the execution of clinical investigations and the initiation of therapy.

Herein, we report a 16-month-old infant experiencing, from 12 months of age onwards, multi-daily paroxysmal episodes of abnormal head postures, predominantly to the right, lasting less than 10 s with spontaneous resolution. This posture is not consistently observed and becomes more pronounced during deambulation (Figure 1).

The patient was born from an uneventful pregnancy and had unremarkable prenatal and perinatal periods. Family history was negative for neurological/gastrointestinal disorders. He experienced physiological jaundice on the third day of life and was treated with 24 h phototherapy. Regular growth and psychomotor development were observed. He was breastfed and weaned regularly. In the two weeks preceding the onset of torticollis, which was concurrent with an infectious episode, there was a reported decrease in appetite.

On evaluation, the child showed normal neurological examination with occasional toe-walking and uncertainty during postural transitions, particularly neglecting the right hemibody. The sternocleidomastoid was normal with the absence of spasms. Moreover, mild dysmorphic features—such as epicanthal folds and a low-set auricular placement—were evident. 

### 2.2. Diagnostic Investigations and Management

Diagnostic work-up included hematological tests (complete blood count, hepatorenal function, coagulation), orthoptic–ophthalmological exam, genetic consultation, brain magnetic resonance imaging (MRI), and somatosensory evoked potentials in the upper limbs, all of which resulted normal. Psychomotor development was assessed using the Griffiths-III [13,14] and revealed a general developmental quotient of 92, with an equivalent developmental age of 15 months, compared to a chronological age of 16 months, placing the child within the average range for his age. During a polysomnographic electroencephalography (EEG), isolated spikes were noted in the occipital region, the pathological significance of which remains uncertain.

Given the diagnostic hypothesis of benign paroxysmal genetic dystonia, an NGS gene panel (including *CACNA1A*, *ATP1A2*, and *SCN1A*) along with direct sequencing of the *PRRT2* gene were performed, both yielding negative results.

Considering the possibility of Sandifer’s syndrome, empiric therapy with a proton pump inhibitor (esomeprazole 0.8 mg/Kg/day) was initiated, with no observed side effects. After a week of treatment, the frequency of paroxysmal episodes completely ceased. 

## 3. Discussion

Sandifer syndrome is a rare neurological disorder characterized by sudden involuntary dystonic postures, including opisthotonus and atypical twisting movements of the head and neck. These episodes typically occur in temporal association with gastroesophageal reflux disease (GERD), making SS a condition closely linked to gastrointestinal dysfunction [2,15].

The pathophysiology of SS remains incompletely understood, but it is thought that the discomfort or pain associated with reflux may trigger these abnormal movements as a compensatory mechanism. In particular, vagal afferent fibers transmit impulses to the NTS, which then connect to the nucleus ambiguus (NA) and nucleus dorsalis nervi vagi (NDX), located in the medullary part of the reticular formation and the medulla oblongata of the brainstem, respectively, ultimately leading to sternocleidomastoid and trapezius muscle contractions and intermittent torticollis [7].

The syndrome was first described in the 1960s [1,3], and since then, the vast majority of reported cases in the literature have involved young neurologically normal children, with no clear sex predilection [16,17].

Torticollis, a prominent feature of SS, is a nonspecific symptom that can present in a variety of clinical contexts. It may manifest as a persistent or paroxysmal condition and often serves as the primary reason for referral across multiple medical specialties. Given the wide differential diagnosis of torticollis, ranging from benign positional abnormalities to more serious neurological and musculoskeletal disorders, it is crucial to undertake a thorough and comprehensive evaluation of all patients who present with this symptom. This careful approach is particularly important as the underlying causes of torticollis can be quite varied, encompassing both congenital and acquired conditions. While GERD is a recognized but rare cause of torticollis, it should be considered in the differential diagnosis, particularly in the pediatric population.

In many cases, the paroxysmal nature of the head twisting episodes is linked to meals, and they may subside with the resolution of the reflux symptoms. However, it is essential to note that in patients with SS, the neurological examination, as well as the evaluation of the sternocleidomastoid and trapezius muscles, typically reveal normal findings [18], distinguishing it from other potential causes of torticollis with more significant neurological involvement, such as cervical dystonia or neurological syndromes.

In contrast to other reports in the literature, the case described in this report does not align with the classic pattern of meal-induced torticollis. In our patient, the paroxysmal torticollis episodes did not correlate with mealtimes or any other identifiable triggers. Furthermore, the patient did not present with the gastrointestinal symptoms commonly associated with GERD, such as abdominal pain, vomiting, or sialorrhea, nor did they exhibit typical psychiatric features such as excessive crying, fussiness, or irritability (Table 1).

This distinction highlights the heterogeneity of Sandifer syndrome presentations and emphasizes the need for clinicians to maintain a broad differential diagnosis, especially in cases where the classic triggers and symptoms are absent.

## 4. Conclusions

It is crucial to recognize the importance of early diagnosis and appropriate management of Sandifer syndrome, particularly in distinguishing it from other causes of torticollis and movement disorders. While the diagnosis of SS remains primarily clinical, based on characteristic dystonic movements associated with GERD, an empiric trial of proton pump inhibitors can be beneficial in suspected cases, helping to avoid invasive diagnostic procedures. This was demonstrated in the present case, where paroxysmal episodes resolved after initiating treatment with esomeprazole. The limitations of our case report include the lack of confirmatory tests for GERD, such as pH impedance or upper gastroendoscopy, due to the invasive nature of these procedures and the very young age of our patient.

## Figures and Tables

**Table 1:** Differences between regular presentation of Sandifer syndrome and our case showing some atypical symptoms.

	Typical Presentation of SS [2,11]	Our Case
Correlation with the meal or other triggers	Exacerbation of the abnormal posturing immediately after eating and improvement in between feeds	No correlation with the meal or any other trigger
Neurological clinical features	Various signs and symptoms such as severe hypotonia, writhing movements of the limb, torticollis, dystonia	Paroxysmal torticollis
Psychiatric clinical features	Excessive crying, fussiness, irritability, choking during and after feeding	No psychiatric features
Gastrointestinal clinical features	Abdominal pain, vomiting, sialorrhea	No gastrointestinal features

## Data Availability

Data regarding this case are available upon reasonable request to the corresponding author.

## Abbreviations

The following abbreviations are used in this manuscript:

SS	Sandifer syndrome
GERD	gastroesophageal reflux disease
PPIs	proton pump inhibitors
MRI	magnetic resonance imaging
EEG	electroencephalography
NTS	nucleus tractus solitarii
NA	nucleus ambiguus
NDX	nucleus dorsalis nervi vagi
NGS	next-generation sequencing

## References

[1] Bray P.F., Herbst J.J., Johnson D.G., Book L.S., Ziter F.A., Condon V.R. (1977). Childhood gastroesophageal reflux: Neurologic and psychiatric syndromes mimicked. JAMA.

[2] Lehwald N., Krausch M., Franke C., Assmann B., Adam R., Knoefel W. (2007). Sandifer syndrome—A multidisciplinary diagnostic and therapeutic challenge. Eur. J. Pediatr. Surg..

[3] Kinsbourne M. (1964). Hiatus hernia with contortions of the neck. Lancet.

[4] Bamji N., Berezin S., Bostwick H., Medow M.S. (2015). Treatment of Sandifer syndrome with an amino-acid-based formula. AJP Rep..

[5] Moore D.M., Rizzolo D. (2018). Sandifer syndrome. J. Am. Acad. Physician Assist..

[6] Mordekar S.R., Velayudhan M., Campbell D.I. (2017). Feed-induced dystonias in children with severe central nervous system disorders. J. Pediatr. Gastroenterol. Nutr..

[7] Cerimagic D., Ivkic G., Bilic E. (2008). Neuroanatomical basis of Sandifer’s syndrome: A new vagal reflex?. Med. Hypotheses.

[8] Kabakuş N., Kurt A. (2006). Sandifer Syndrome: A continuing problem of misdiagnosis. Pediatr. Int..

[9] Bayram A.K., Canpolat M., Karacabey N., Gumus H., Kumandas S., Doğanay S., Arslan D., Per H. (2016). Misdiagnosis of gastroesophageal reflux disease as epileptic seizures in children. Brain Dev..

[10] Sondheimer J.M., Morris B.A. (1979). Gastroesophageal reflux among severely retarded children. J. Pediatr..

[11] Kato D., Uchida H., Amano H., Yokota K., Shirota C., Tainaka T., Sumida W., Makita S., Yasui A., Gohda Y. (2024). A systematic review of Sandifer syndrome in children with severe gastroesophageal reflux. Pediatr. Surg. Int..

[12] Mindlina I. (2020). Diagnosis and management of Sandifer syndrome in children with intractable neurological symptoms. Eur. J. Pediatr..

[13] Green E., Stroud L., O’Connell R., Bloomfield S., Cronje J., Foxcroft C., Hurter K., Lane H., Marais R., Marx C. (2016). Griffiths Scales of Child Development.

[14] Lanfranchi S., Rea M., Ferri R., Vianello R. (2019). Studio di Validazione e Standardizzazione Italiana Delle Griffiths III.

[15] Sauer C.G., Kugathasan S. (2010). Pediatric inflammatory bowel disease: Highlighting pediatric differences in IBD. Med. Clin. N. Am..

[16] Corrado G., Cavaliere M., ‘Eufemia D. (2000). Sandifer’s syndrome in a breast-fed infant. Am. J. Perinatol..

[17] Shahnawaz M., van der Westhuizen L.R., Gledhill R.F. (2001). Episodic cervical dystonia associated with gastro-oesophageal reflux. A case of adult-onset Sandifer syndrome. Clin. Neurol. Neurosurg..

[18] Tekou H., Foly A., Akue B., Senah K.C., Dagnrah P.C., Atanley R. (1998). Le syndrome de Sandifer. Tunis. Médicale.

